# Changes in professional commitment of undergraduate nurse students before and after internship: a longitudinal study

**DOI:** 10.1186/s12909-022-03364-0

**Published:** 2022-04-14

**Authors:** Ling Zhao, Yinhua Su, Na Jiang, Fanhua Zhou, Li Liao, Yannan Liu

**Affiliations:** 1 University of South China School of Nursing, West Changsheng Road #28, Hengyang, 421001 Hunan Province China; 2 Xiangnan University School of Nursing, Chenzhou, Hunan Province China; 3 School of Nursing, Hunan University of Medicine, Huaihua, Hunan Province China

**Keywords:** Professional commitment, Perceived stress, Internship, Nursing students, Clinical instructors

## Abstract

**Background:**

Experiencing internship shapes nursing students’ professional commitment and aggravates its changes. However, few studies have been investigated how this changes empirically.

**Objectives:**

The aims of this study are to investigate (a) what are the changes of professional commitment of nursing students before and after the internship? (b) Which of multiple independent variables is the strongest predictor?

**Methods:**

A longitudinal study was conducted with 996 senior undergraduate nursing students (ready to enter clinical practice) in the China universities. The survey was conducted in the spring of 2015 and autumn of 2016. The data were collected by a paper-and-pencil questionnaire. The instruments used included Professional Commitment Scale and Perceived Stress Scale. Analysis of paired *t*-test and linear regression analysis were performed on the data.

**Results:**

Nursing students showed lower professional commitment (2.79 ± 0.36) than they were (2.92 ± 0.36) before internship. Socio-demographic variables, pre-internship professional commitment and stress perceived during internship predicted 40.1% of the variance in the post-internship commitment.

**Discussion:**

These data summarize the nursing students’ professional commitment changes and the main influential factors that contribute to post-internship professional commitment of undergraduate nursing student. The findings are timely, which indicate that senior nursing students’ professional commitment can be increased by enhancing pre-internship commitment and reducing students’ stress levels during internship.

## Background

Professional commitment is defined as an attitude that provides a physical, mental and emotional connection to one’s work. It is also the harmony between an individual’s beliefs and their determination to continue working in their profession. It is composed of three factors: a belief in the goals and values of the profession, a willingness to make an effort to understand these values and a determination to stay in one’s profession [1–3]. Higher professional commitment among nurses can reduce turnover intention, and improve professional competence, job satisfaction and the quality of patient care [4–6]. Nursing students who possess higher professional commitment levels during their college years are believed to have improved professional commitment levels when they become registered nurses after graduation [7]. The future of nursing will be determined by nurses and nursing students who are committed to the profession [8].

Level of professional commitment has an important influence on a nursing student’s’ career decision-making [9]. Studies have shown that lower professional commitment affects nursing students’ work values [10, 11]. Lower professional commitment is also a negative indicator of job burnout and a desire to leave the profession [12]. To our disappointment, Chinese nursing students have shown only a moderate level of professional commitment during their undergraduate year [13]. Empirical investigations even show a reduced level of professional commitment among senior nursing students. For example, Tan investigated 663 undergraduate nursing students and found that senior-year students had lower professional commitment than students in grades [14]. This finding has been supported by other studies [15, 16]. This finding signals that nursing students’ professional commitment deserves greater attention from educators and scholars. Particularly senior nursing students should be examined, along with the clinical practice of their nursing education, which is the transition period from their safe and familiar university study environment to the real clinical environment.

The clinical environment plays a crucial role, especially in the clinical training of nursing students, as they come into contact with the realities of their function and form opinions on their professional careers [17]. According to person-environment fit theory, the development of professional commitment occurs as a result of the fit between individuals and their profession [18]. When a match exists between individuals and their environment, favorable outcomes result. Fan et al. found that nursing students’ satisfaction with clinical practice, the degree of nursing students be valued by hospital and nurse students’ satisfaction with instructors can affect nurse students’ professional commitment [19]. Therefore, Therefore, we argue that nursing students will have expectations for clinical practice; if they meet their expectations, they will be satisfied with their clinical practice, and they will conclude that they are suitable for the job.

Nursing students frequently experience various stressors in the clinical setting [2]. Pressure is multifaceted, such as the stress and difficulties one may experience due to a misfit between one’s previous college studying experience and a reality framed by increased complexity of interpersonal relationships, lack of adequate clinical skills and high workloads [20]. Tensions with clinical instructors and patients, lack of understanding and trust from patients and their family, and poor rewards can create disappointment among nursing students [21, 22]. They are all related to negative professional perception [19, 23].

Clinical practice is an essential part of nurse training that plays a crucial role in shaping professional attitude, professional emotion and professional identity [24]. In China, undergraduate nursing students are required to spend their full-time practice in hospitals in their 3rd to 4th academic year. Undergraduate nurse students will act as expectant registered nurses to work in the hospital, and their main task is to apply their knowledge and skills, which have been taught in school, to clinical practice. Each nursing student is supervised by an experienced clinical instructor. For graduates in clinical practice, their main task is to search for scientific questions and resolve them. There is some concern about changes in students’ commitment before and after internships. A study of 115 nursing students at a tertiary hospital found that students’ professional commitment increased after the internship [25]. There has been little empirical research on changes in the professional commitment of nursing students before and after internships, let alone exploration of the main factors underlying variability in professional commitment. To fill this gap, in this study, two research questions were investigated:

1. What are the changes in the professional commitment of nursing students before and after the internship?
2. Which of multiple independent variables is the strongest predictor of changes in professional commitment?

## Methods

### Study design

This was a longitudinal study of nurse students directed by a team of nursing and health researchers. The study was carried out in association with nursing students from several universities in Hunan, China. The study received ethics committee approval from the University of South China. Written consent was obtained from the participants in this study after obtaining informed consent.

### Sampling

Cluster sampling was used in this study. First, the universities were divided into first-batch, second-batch, and newly approved second-batch universities. We chose one university from each type of university. Participants who met this inclusion criterion were invited to participate in this study. Nursing students were eligible if they 1) were aged between 18 and 30 years, 2) spoke/read Chinese, 3) were enrolled in the Bachelor of Science in Nursing Program, and 4) started internship during the spring of 2015 and ended their internship during the autumn of 2016. Participants who failed to complete the 10-month internship were removed from the sample.

### Data collection

**Fig. 1:**
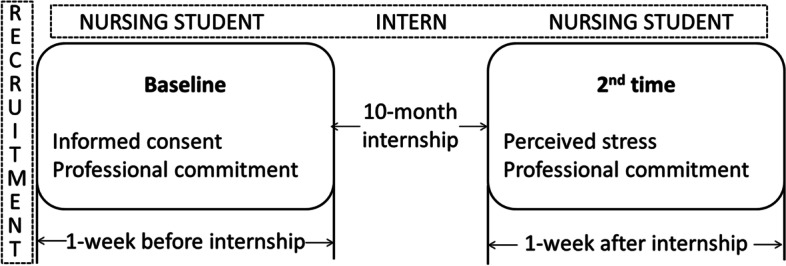
Schematic of the study design

The data were collected by the researchers between 10 April 2015 and 20 August 2016 from three universities located in three cities in China. To conduct the data collection process, researchers personally visited the universities. First, we shared some information about professional commitment to inform students of its importance to their future careers. Then, we solicited their participation with the following question: Do you want to know whether and how your professional commitment will change from before to after your internship? Finally, we informed the participants of our study and obtained written consent from those who agreed to participate. Data were collected at two time points. Participants were recruited via in-class information sessions. Baseline measurements were conducted for 1 week before students entered the internship. The second measurement was performed within 1 week after the internship (Fig. 1). The questionnaire was distributed during a lecture, and each student was given 20 min to complete it during the lecture. Confidentiality was reassured by keeping the submitted raw data in an encrypted file and computer. Among the 1250 contacted nursing students, 996 responded, for a response rate of 85.9%. Compared with the average response rate of 52.7%, the rates of this study achieved a satisfactory level [26].

### Instruments

The Professional Commitment Scale, developed and revised by Lu, is often used to test nurses’ and nursing students’ professional commitment levels [3, 27]. In the original version, Cronbach’s α value was 0.93. It consists of 23 items and three subscales, which are as follows: willingness to make an effort (9 items), maintaining membership (8 items), and belief in goals and values (6 items). All questions were scored on a 4-point scale, from 1 (strongly disagree) to 4 (strongly agree), with higher scores indicating stronger professional commitment. In this study, Cronbach’s α was 0.88, indicating strong internal consistency.

The Perceived Stress Scale was developed to assess the types of stressful events and the degree of stressors that occurred during clinical practice. It consists of 6 subscales using 29 items to assess stress from taking care of patients (8 items), stress from teachers and nursing staff (6 items), stress from assignments and workload (5 items), stress from peers and daily life (4 items), stress from lack of professional knowledge and skills (3 items), and environmental stress (3 items). All items used a 5-point rating scale ranging from 0 (never) to 4 (always), with good internal consistency (α = 0.89). The Chinese version also showed good internal consistency (α = 0.90) [28]. In this study, its internal reliability reached a satisfactory level, with a Cronbach’s α of 0.90.

### Data analysis

The data were recorded and analyzed using IBM SPSS version 23.0. Descriptive statistics were used to describe demographic variables, commitment and perceived stress levels. A paired *t test* was performed to compare pre- and post-internship commitment. Cohen’s *d* was used to assess standardized differences for the comparison between two means. Linear regression analysis (a univariate regression analysis first and then a multivariate analysis, using a stepwise method) was performed to explore the effect of independent variables (such as demographic variables, pre-internship commitment, and perceived stress in clinical practice) on nurse students’ professional commitment after internship. Goodness-of-fit was evaluated using R-squared values.

## Results

The majority of the participants were female (955; 95.9%). The mean age of the students was 20.83 years (SD 1.03). One hundred thirty-eight students completed their internships in tertiary hospitals. Most of the students’ families (50.8%) lived in rural areas with moderate or poor economic levels. The sociodemographic characteristics of the students are displayed in Table 1.

**Table 1:** Participants characteristics (*N* = 996)

Demographics Characteristic	Frequency (%)
Gender, female	955 (95.9)
Only child in the family	
Yes	199 (20.0)
No	797 (80.0)
Family address	
Country	506 (50.8)
Town	185 (18.6)
City	305 (30.6)
Family economy	
Poor	339 (34.0)
Moderate	412 (41.4)
Rich	245 (24.6)
Being awarded scholarship	
Yes	410 (41.2)
No	586 (58.8)
Class leader	
Yes	494 (49.6)
No	502 (50.4)
Course arrangement	
Dissatisfied	4 (0.4)
Not very satisfied	108 (10.8)
Quite satisfied	584 (58.6)
Satisfied	300 (30.1)
Interest in nursing	
Yes	727 (73.0)
No	269 (27.0)
Feeling for being a nurse	
Not good	56 (5.6)
No feelings	103 (10.3)
Good	837 (84.0)
Practical hospital meet expectation	
Yes	747 (75.0)
No	249 (25.0)
Satisfied for clinical internship	
Dissatisfied	8 (0.8)
Less satisfied	92 (9.2)
More satisfied	515 (51.7)
Satisfied	381 (38.3)

A paired *t test* was conducted to investigate whether there were differences in professional commitment before and after the internship among nursing students. The degrees of freedom in the t test statistics was 995. Before the internship, the participants’ mean Professional Commitment Scale total, Willingness to make an effort subscale, Maintaining membership subscale, and Belief goals and values subscale scores were 2.92 ± 0.36, 2.88 ± 0.39, 2.90 ± 0.37, and 2.99 ± 0.49, respectively. After internship, the participants’ mean Professional Commitment Scale total, Willingness to make an effort subscale, Maintaining membership subscale, and Belief goals and values subscale scores were 2.79 ± 0.36, 2.77 ± 0.45, 2.81 ± 0.40, and 2.79 ± 0.40, respectively. The Cohen’s *d* values representing changes in pre- and post-internship professional commitment scale total scores, Willingness to make an effort subscale scores, Maintaining membership subscale scores, and Belief goals and values subscale scores were 0.351, 0.250, 0.225, 0.400, respectively. The results showed that there were significant differences in the scores for overall commitment, willingness to make an effort, maintaining membership, belief goals, and values (*t* = 11.205, *P* = 0.000, *t* = 7.847, *P* = 0.000, *t* = 7.330, *P* = 0.000, *t* = 12.122, *P* = 0.000, respectively), which means that there was significant change in commitment from pre- to post-internship (Table 2).

**Table 2:** Paired *t*- test between pre- and post-internship professional commitment (*N* = 996)

Variables	Pre-internship (Mean ± SD)	Post-internship (Mean ± SD)	*t-*test	*P*	*CI*	
					lower	upper
Overall commitment	2.92 ± 0.36	2.79 ± 0.36	11.205	0.000	0.108	0.154
Willingness to make an effort	2.88 ± 0.39	2.77 ± 0.45	7.847	0.000	0.082	0.137
Maintaining membership	2.90 ± 0.37	2.81 ± 0.40	7.330	0.000	0.068	0.118
Belief goals and values	2.99 ± 0.49	2.79 ± 0.40	12.122	0.000	0.160	0.222

Table 3 shows the analysis of sociodemographic factors and professional commitment. The female nursing students who were very much interested in nursing and those who had good feelings about being a nurse showed the greatest increase from pre- to post-internship professional commitment, with a significant *P* value< 0.05.

**Table 3:** Analysis of demographic factors and professional commitment (*N* = 996)

Variables	Pre-internship			Post-internship		
	*B*	*t*	*P*	*B*	*t*	*P*
Gender	−0.217	−4.786	0.000	−0.142	− 2.815	0.005*
Age	−0.015	−1.561	0.119	−0.061	−5.668	0.000*
Only child in the family	0.056	2.071	0.039	0.016	0.534	0.594
Family address	−0.024	−1.958	0.051	−0.012	−0.856	0.392
Class leader	0.053	2.781	0.006	0.033	1.542	0.123
Interest in nursing	0.211	9.292	0.000	0.156	6.162	0.000*
Feeling for being nurse	0.227	11.799	0.000	0.116	5.381	0.000*

*Significance level was accepted as *P* < 0.05

Some variables, such as only child in the family (*P* = 0.225), family address (*P* = 0.318), and class leader (*P* = 0.489), were excluded from the multiple regression analysis. Multiple regression analysis was performed with post-internship commitment as the dependent variable, while demographic information, pre-internship commitment, and perceived stress were entered as independent variables to explore the main factors affecting post-internship commitment. The model explained 40.1% of the variation in post-internship commitment (F = 56.407, *P* < 0.001). The results of the regression analysis showed that the most significant factor associated with post-internship commitment was pre-internship commitment (*β* = 0.362, *P* < 0.05), followed by Stress from taking care of patients (*β* = − 0.170, *P* < 0.05), Stress from teachers and nursing staff (*β* = − 0.169, *P* < 0.05), Satisfied with course arrangement (*β* = 0.159, *P* < 0.05), Satisfied with internship (*β* = 0.133, *P* < 0.05), Being awarded a scholarship (*β* = 0.133, *P* < 0.05), Environmental stress (*β* = 0.105, *P* < 0.05), Hospital practicum met expectations (*β* = 0.102, *P* < 0.05), Stress from lack of professional knowledge and skills (*β* = 0.091, *P* < 0.05), Family financial status (*β* = − 0.061, *P* < 0.05) and Gender (*β* = − 0.052, *P* < 0.05) (Table 4). The regression equation is as follows:

**Table 4:** Multiple regression results: predictors for post-internship commitment (*N* = 996)

Variables	*B*	SE	*β*	*t*	*P*	*95%CI*
Constant	1.309	0.113		11.536	0.000	1.086 to 1.531
Pre-internship professional commitment	0.368	0.027	0.362	13.758	0.000	0.361 to 0.421
Satisfied for course arrangement	0.092	0.015	0.159	5.976	0.000	0.062 to 0.123
Stress from taking care of patients	−0.110	0.025	−0.170	−4.499	0.000	−0.159 to − 0.062
Satisfied for internship	0.074	0.015	0.133	4.830	0.000	0.044 to 0.104
Being awarded scholarship	0.093	0.019	0.126	5.045	0.000	0.057 to 0.130
Practical hospital meet expectation	0.086	0.022	0.102	3.831	0.000	0.042 to 0.129
Stress from teachers and nursing staff	−0.127	0.028	− 0.169	−4.528	0.000	−0.182 to − 0.072
Stress from lack of professional knowledge and skills	0.067	0.023	0.091	2.846	0.005	0.021 to 0.113
Stress from the environment	0.080	0.028	0.105	2.856	0.004	0.025 to 0.13.5
Family economy	−0.029	0.012	− 0.061	−2.483	0.013	− 0.053 to − 0.006
Gender	− 0.086	0.042	− 0.052	− 2.036	0.042	− 0.003 to − 0.158

Durbin-Watson = 1.722; *F* = 56.407, *P* < 0.05; R = 0.639; R^2^ = 0.408; AdjustedR^2^ = 40.1%

*Abbreviations*: *CI* Confidence interval, *SE* Standard error, *β* Standardized regression coefficient

Post-internship commitment = 1.309 + 0.368 × Preinternship commitment+ 0.092 × Satisfied with course arrangement-0.110 × Stress from taking care of patients+ 0.074 × Satisfied with internship+ 0.093 × Being awarded a scholarship+ 0.086 × Hospital practicum met expectations-0.127 × Stress from teachers and nursing staff+ 0.067 × Stress from lack of professional knowledge+ 0.080 × Environmental stress-0.029 × Family financial status-0.086 × Gender.

## Discussion

The primary purpose of this study was to explore the changes in the professional commitment of nursing students in China before and after their internships. In this study, nursing students’ post-internship commitment was indeed lower than their pre-internship commitment. This finding is not consistent with those of a previous study. A recent study by Sultan et al. reported that after the nursing internship, the professional level of students increased. They investigated 101 senior nurse students in Turkey [29]. The different results may be attributed to the different learning styles and practice experiences between China and Turkey. A study by Bobby also reported increased professional commitment levels among nursing students. In that study, 115 students were investigated in the same hospital in China, and most of them were diploma-level nursing students [25]. This may in part account for the different results. Clinical practice can serve as a bridge between theory and practice, which is a necessary process for training competent professional nurses [30, 31]. It has a strong relationship with nursing professionalism and identity [32]. The present study found that stressful clinical practice may have a negative impact on nursing students’ professional commitment. This may be explained by the idea that students usually start out with an idealistic view of the profession, which is then corrected when they have a more accurate view of what the job involves [33]. Previous studies have shown that nursing students generally have high expectations for internships [34]. Students in this study had a bachelor’s degree, which shows that the higher their education level is, the higher their expectations. It is true that there is a great discrepancy between school-based and hospital-based learning for nursing students, such as new knowledge and skills, the sequencing of theory and practice, and teachers’ teaching roles, which can lead to frustration [35–37]. Studies have also found that factors such as lack of recognition and understanding, greater responsibility, poor working conditions, limited career opportunities, subordination to orders and low status can also lead to nursing students’ negative attitudes toward the profession during the internship [38]. High expectations for internships, frustration resulting from theory-practice gaps and negative attitudes during internships may contribute to a decreased commitment after internships.

A negative environment, such as gender bias, may account for differences in professional commitment between male and female nursing students [39]. In this study, those nursing students who were very much interested in nursing and those students who had a good feeling about being a nurse demonstrated the greatest increase from pre- to post-internship professional commitment, which indicates that nursing students’ interest and professional conception may shape their professional commitment. Nursing students who are interested in nursing and have good feelings about being a nurse may easily achieve good individual experiences and self-image. The congruence between person and environment results in stronger commitment [40]. Their interest and good feeling for being a nurse encouraged them to think that they were suited to the profession. This no doubt can increase students’ professional commitment levels.

The second aim of this study was to explore which of multiple independent variables was the strongest predictor of post-internship commitment. In this study, sociodemographic variables, pre-internship professional commitment and perceived stress accounted for 40.1% of the variance in post-internship professional commitment (Table 4). According to this result, nursing students’ post-internship professional commitment was affected by numerous factors, of which pre-internship commitment was the strongest predictor, indicating that students with higher pre-internship professional commitment also maintained higher commitment after internship. In a previous study, fewer studies focused on the linkage between pre- and post-internship commitment. Only a few studies have compared the differences between them, albeit using small samples and focusing in just one area [25, 29]. Students’ professional commitment is an ongoing, dynamic process that starts on the first day of university education [41]. There is a constant, active relationship between pre-internship professional commitment and post-internship professional commitment [29].

The second variable in order of importance was “Stress from taking care of patients”; that is, as the stress from taking care of patients decreased, their professional commitment level increased. Patient attitudes can also affect a student’s professional commitment; the professional commitment of nursing student entering internship was influenced by patients’ recognition [42]. Nursing students’ development of professional commitment must be built through patients’ recognition. When they feel not trusted or unaccepted by patients and are therefore unable to reach expectations, there is no equilibrium between the give and the take, which is bound to shake professional conviction and negatively influence professional commitment.

The third variable, in order of importance, for predicting the Professional Commitment Scale score, according to regression analysis, was “the stress from teachers and nursing staff.” Nursing students who experienced less stress from teachers and nursing staff had higher professional commitment levels than those who experienced higher stress from teachers and nursing staff (Table 4). Previous research has shown that nursing students’ professional identity is easily affected by clinical teachers [43]. In China, nurses who act as clinical instructors have to face busy clinical jobs; because of time constraints, they do not have enough time and energy to guide nursing students. Students feel a lack of care and guidance from teachers, which scored highest in our study (2.51 ± 0.67); this no doubt will increase nursing students’ stress. Perceived support from nursing teachers was positively correlated with students’ professional identity [42]. When students felt less guidance and support from their clinical instructors and nursing staff, this negatively affected their post-internship professional commitment.

The fourth variable in order of importance was “Satisfied with course arrangement.” As the nursing students’ satisfaction with course arrangement increased, so too did their professional commitment level. Bobby’s study indicated that course arrangement satisfaction was positively related to the nurse self-concept [25]. There are several reasons why satisfaction with course arrangement would be associated with students’ professional commitment levels. First, satisfaction with course arrangement increases nurse students’ nurse self-concept, and research shows that a sufficient nurse self-concept can lead to successfully fulfilling the nursing role [44]. Second, a higher professional self-concept was associated with a reduction in the level of stress, which will help students to be better suited for clinical internships [45].

The fifth variable in order of importance was “Satisfied with internship.” Nursing students’ professional identity is the result of a process that emerges through the interaction between neophyte, university teacher and tutor during the internship [46]. Satisfaction with internship implies a good experience during clinical practice, which likely encourages nursing students to think that they are suitable for the nurse role. The match between role and environment thus enhances their professional commitment level.

The following variables in order of importance were “Hospital practicum met expectations,” “Environmental stress” and “Stress from lack of professional knowledge and skills.” There was a close connection between “hospital practicum met expectations” and “satisfaction with internships.” Together with students’ experience, they shape their professional commitment. The literature showed that there was a clear gap between the content taught in school and the clinical practical experience of nursing students [47–50]. The discrepancies between theoretical education and nurses’ performance within clinical settings lead to incompatibility within the clinical environment and a feeling of incapability and uselessness of their knowledge. Most studies have found that the lack of professional knowledge and skill is the main source of stress within clinical practice [51, 52]. The complex and ever-changing hospital environment also adds great stress to nursing students due to a lack of professional knowledge. The less professional knowledge they have, the more difficult it is for them to care for patients, which leads to reduced self-confidence and increased frustration. The complex environment plus the stress from a lack of knowledge and skill increases role ambiguity. In addition, nursing students with role ambiguity have a more fragile and unstable professional identity, which can undermine commitment [53].

As shown in our study, students experienced decreased professional commitment levels after the clinical internship. Therefore, we should take some measures to prevent declines in professional commitment following clinical practice. There was a significant association between pre- and post-internship professional commitment. Thus, nursing education should reinforce senior nursing students’ professional commitment during university education. Furthermore, perceived stress in internships was associated with post-internship professional commitment. Institutions and hospital managers should focus on the perceived stress of nursing students in internships and formulate some strategies to reduce their stress levels.

### Limitations

As this study was carried out in three universities located in different cities in China, the study results may lack external validity. Moreover, the data were collected during a lecture, so absent students might have had different responses on the instruments. Other data collection methods, such as mailing out surveys, may be considered in the future. The results of this study may have been affected by factors such as time, clinical practice setting and number of nursing students in the three universities where the sample was selected. To minimize the effect of these factors, future studies should broaden sample groups and incorporate different time points to evaluate stress levels and professional commitment. A strength of this study is that it is the first to describe changes in professional commitment among a large group of Chinese undergraduate nursing students before and after their internships. Another strength of this study is that this is the first to explore the strongest predictor of nursing students’ post-internship professional commitment levels using multiple regression.

## Conclusion

In our study, we reported that the professional commitment levels of nursing students after internship were lower than those before clinical practice. This study determined that the main factors affecting senior nursing students’ professional commitment were pre-internship professional commitment, “Stress from teachers and nursing staff,” “Stress from taking care of patients,” satisfaction with internship, “Hospital practicum met expectations,” “Environmental stress” and “Stress from lack of professional knowledge and skills.”

## Data Availability

Not applicable.
